# Diabetes care among elderly medicare beneficiaries with Parkinson’s disease and diabetes

**DOI:** 10.1186/s40200-015-0209-3

**Published:** 2015-10-05

**Authors:** Sandipan Bhattacharjee, Usha Sambamoorthi

**Affiliations:** Department of Pharmacy Practice and Science, School of Pharmacy, The University of Arizona, 1295 North Martin Avenue, Tucson, 85721 AZ USA; Department of Pharmaceutical Systems & Policy, School of Pharmacy, West Virginia University, Morgantown, WV USA

**Keywords:** Parkinson’s disease, Co-occurring conditions, Standards of Care, Propensity Score

## Abstract

**Background:**

Elderly individuals with type 2 diabetes mellitus (T2DM) suffer from several comorbidities, which affect their health outcomes, as well as process of care. This study assessed process and intermediate clinical outcomes of diabetes care among elderly individuals with T2DM and co-occurring Parkinson’s disease(PD).

**Methods:**

This study used a retrospective cohort design with propensity score matching using Humana Medicare Advantage Part D claims database (2007-2011) and included elderly (age ≥ 65 years) Medicare beneficiaries with T2DM (identified by ICD-9-CM code of 250.x0 or 250.x2). PD was identified using ICD-9-CM code of 332.xx. After propensity score matching there were 2,703 individuals with T2DM and PD and 8,109 with T2DM and no PD. The three processes of care measures used in this study included: (i) HbA1c test; (ii) Lipid test; (iii) and Nephropathy screening. Intermediate clinical outcomes consisted of glycemic and lipid control.

**Results:**

Multivariable conditional logistic regressions revealed that elderly individuals with T2DM and PD were 12 % (AOR: 0.88, 95 %CI: 0.79-0.97) and 18 % (AOR: 0.82, 95 %CI: 0.72-0.94) less likely to meet the annual American Diabetes Association (ADA) recommended HbA1c and lipid testing goals respectively compared to individuals with T2DM and no PD. Multinomial conditional logistic regressions showed that elderly individuals with T2DM and PD were more likely to have HbA1c and lipid (HbA1c < 8 %; LDL-C <100 mg/dl; HDL-C ≥ 50 mg/dl; triglyceride <150 mg/dl; and total cholesterol <200 mg/dl) control.

**Conclusions:**

Among elderly individuals with T2DM, those with PD were less likely to achieve ADA recommended annual HbA1c and lipid testing compared to those without PD. However, PD individuals were more likely to achieve intermediate glycemic and lipid control.

## Introduction

Type-2 Diabetes Mellitus (T2DM) and its complications are the leading causes for morbidity and mortality in the United States (U.S.) [1]. Parkinson’s disease (PD) is a progressive neurodegenerative disease characterized by muscular tremor, slowing of movement, partial facial paralysis, peculiarity of gait and posture [2, 3]. Individuals with co-occurring T2DM and PD can have poor quality of life, impaired health and functional status. Moreover, co-occurring T2DM and PD require medical care from different specialties such as neurologists and endocrinologist, and require care in different settings including home-based as well as facility-based care [4]. Co-occurring T2DM and PD can pose significant challenges to diabetes management.

However, clinical guidelines for diabetes management for those with T2DM and PD are lacking. In general, guidelines for clinical practice are developed based on the expert consensus and the scientific evidence for a single disease state [5]. Standards of care and quality of care improvement efforts are based on these guidelines for clinical practice for individual disease state. As for example, there are separate guidelines for treating diabetes (such as the guidelines of the American Diabetes Association) and PD (European Parkinson’s disease Standards of Care Consensus Statement).

Studies on diabetes care among individuals with other co-occurring chronic conditions have revealed mixed findings. One study conducted among veterans with diabetes seeking care in seven different Veterans Affairs (VA) facilities from July 2007 through June 2008 reported that veterans with T2DM were more likely to receive overall good quality for all three quality measures (glycemic, blood pressure and low density lipoprotein-cholesterol control) combined (adjusted OR, 2.17; 95 % CI, 1.96–2.39) [6]. Whereas, another study using the INTERMED classification system for case complexity found that greater complexity (defined as higher INTERMED scores) among individuals with T2DM was associated with higher HbA1c values [7]. Therefore, it is not known whether clinical complexity is associated with poor or better outcomes among individuals with T2DM.

Despite there being no separate guideline recommendations regarding diabetes process of care (e.g., bi-annual HbA1c testing) in the presence of neurodegenerative conditions, it can be perceived that meeting at least the usual diabetes process of care recommendations is critical to providing appropriate care to elderly individuals with T2DM and PD. However, to the best of our knowledge, no prior study have explored the diabetes process of care among elderly individuals with co-occurring T2DM and PD. The results from the current study will fill a critical knowledge gap regarding the current standards of care among individuals with co-occurring T2DM and PD.

A study of diabetes management among elderly with T2DM and PD is important for several reasons. It has been estimated that by 2030, the prevalence of PD will increase by approximately two-folds due to the aging U.S. population [8] and will become a major public health concern in near future. Moreover, existing literature suggests that the presence of T2DM aggravates neurodegeneration and therefore there is a need of controlling T2DM to prevent further deterioration [9–11]. Hence, the primary aim of this study is to assess the process and intermediate clinical outcomes of diabetes care among elderly individuals with T2DM and PD compared to those with T2DM and no PD.

## Methods

### Study design

A retrospective cohort design with matched case–control approach was used for the purposes of this study. Those with T2DM and PD were considered as cases and those with T2DM and no PD were considered as controls. Controls were selected using propensity score method, which adjusted for gender, age, and diabetes complications severity index (DCSI). The algorithm developed by Chang and colleagues was used to measure the severity of diabetes using DCSI [12]. One case was matched to three controls based on 8 to 1 GREEDY matching technique using propensity score. For 8 to 1 GREEDY matching, the cases and control with same propensity score till the 8^th^ digit are matched, and if they do not match on 8 digits, then it goes to 7-digit matching and so on. The GREEDY matching technique employs a sample without replacement algorithm and if there are more than one matches, then selection of control becomes random. Similar approach for propensity score matching have been used in existing literature to minimize the effect of bias and confounding [13]. Controls were selected from the same calendar year as the cases; if individuals were identified with PD in 2008, all controls were from 2008. If elderly Medicare beneficiaries did not have PD in the initial years and then developed it later during the study time period, they were removed from the control pool.

Elderly individuals with T2DM were followed for a period of 24 months with 12-month baseline period and 12 month follow-up period. Baseline period was defined based on the identification of T2DM and PD. For example, if T2DM and PD cases were identified in 2007, 2007 served as the baseline year, and 2008 served as the follow-up period. Process of care and intermediate clinical outcomes were measured during the follow-up year (i.e., 2008).

#### Data source

The Humana Medicare Advantage Part D database (MAPD) from January 01, 2007 through December 31, 2011 was used for this study. The Humana claims database consists of more than 12 million current and previous enrollees among which approximately 2 million enrollees are from MAPD plans. This study used medical, prescription, laboratory claims and person enrollment summary files. The medical claims contained information related to the type of plan, treatment date, type of admission (trauma, elective, emergency etc.), inpatient length of stay, diagnosis and procedural codes, and total Medicare allowable charges associated with each claim. Prescription claims included information on prescription fill date, medication dispensed, quantity of medication dispensed, net amount paid by Humana and out-of-pocket costs for enrollees. The laboratory Claims contained information on lab test identifying codes, lab results and abnormal value indicator. However, laboratory results are available only for approximately 30 % of the laboratory claims. The patient enrollment summary file included information on the MAPD enrollees’ age, sex, race/ethnicity, and enrollment dates.

### Study population

The study population consisted of elderly Medicare beneficiaries (≥65 years) with T2DM. Elderly Medicare beneficiaries with T2DM were identified by the presence of a minimum of one inpatient or two outpatient visits (at least of 30 days apart) with a primary or secondary diagnosis of T2DM [International Classification of Diseases 9^th^ Modification (ICD-9-CM) code: 250.x0 or 250.*x*2] [14].

#### Inclusion criteria

Other inclusion criteria were: (i) continuous enrollment of 24 months (12-month baseline and 12-month follow-up); and (ii) receipt of at least one oral antidiabetic drug (OAD) or insulin during the baseline year.

#### Dependent variables

##### Process of care

The three processes of care measures used in this study included: (i) HbA1c testing; (ii) lipid testing; and (iii) nephropathy screening. These measures were considered to meet the American Diabetes Association (ADA) guidelines if: (1) HbA1c testing was conducted at least two times a year with a gap of at least three months; (2) lipid testing was conducted at least once a year; and (3) nephropathy screening was conducted at least once a year [15]. A detailed description of the CPT and HCPCS codes are provided in Appendix.

## Intermediate clinical outcomes

### Glycemic control

HbA1c > 9 % represents poor glycemic control and is considered to be a poor performance marker among all elderly individuals with T2DM [16]. One study has used HbA1c < 7 % as representative of optimal glycemic control [16]. However, due to the risk of hypoglycemia, less stringent criteria of HbA1C < 8 % is often considered as acceptable glycemic control among elderly individuals with comorbid conditions, or long standing diabetes or multiple medication use [17]. Therefore, glycemic control outcomes were classified into two groups based on HbA1c values as follows: (i) < 8 % and (ii) ≥ 8 % only among those with available HbA1c values during follow-up year (*N* = 4,983).

### Lipid control

Lipid control outcomes were based on Low Density Lipoprotein Cholesterol (LDL-C), High Density Lipoprotein Cholesterol (HDL-C), triglycerides, and total cholesterol. These lipid control outcomes were categorized based on the American Diabetes Association guidelines [15]. LDL-C was categorized as follows: (i) <100 mg/dl and (ii) ≥ 100 mg/dl. HDL-C was categorized into two for both men and women as follows: (i) ≤ 50 mg/dl; and (ii) > 50 mg/dl. Triglycerides were classified into: (i) <150 mg/dl; and (ii) ≥ 150 mg/dl. Total cholesterol was divided into groups as follows: (i) < 200 mg/dl; and (ii) ≥ 200 mg/dl. Again these were restricted to individuals with available LDL-C (*N* = 2,497), HDL-C (*N* = 4,833), total cholesterol (*N* = 2,543) and triglycerides (*N* = 2,535) values during the follow-up year.

### Key independent variable: presence of PD

The key independent variable for all analyses was presence or absence of PD. Identification of PD was achieved by using ICD-9-CM code of 332.xx during the baseline year. The diagnosis of PD was ascertained by the presence of at least one inpatient or two outpatient visits (30 days apart) with a primary or secondary diagnosis of PD (ICD-9-CM code: 332.xx) [18].

### Independent variables

#### Patient complexities

According to the American Geriatric Society (AGS) guidelines, individuals having specific conditions such as cognitive impairment, depression, fall and falls risk, polypharmacy, and urinary incontinence should be provided individualized treatment [19]. These characteristics were measured during the baseline period. Elderly Medicare beneficiaries were considered to have cognitive impairment due to physical illnesses if they had a diagnosis of Huntington’s disease, delirium, dementia, amnestic and other cognitive disorders; whereas if they have a diagnosis of bipolar disorder, schizophrenia and other psychotic disorders, they were considered to have cognitive impairment due to mental illnesses. Elderly Medicare beneficiaries were considered to have any cognitive impairment if they had either mental and/or physical cognitive impairment. To identify accidental falls, E-codes E880 through E888 were used whereas V-code V15.88 was used as a proxy measure for falls risk [20]. Number of therapeutic classes of prescribed medications was used to define polypharmacy, and was categorized into quintiles: (i) 0–0, (ii) 1–1, (iii) 2–3, (iv) 4–5, and (v) 6–31.

#### Dominant comorbid conditions

Using the framework of Kerr and Piette, cancer, end stage renal disease, and end stage liver disease were included as a dominant comorbid condition in this study [21].

#### Other independent variables

The current study adapts elements from the Vector model of Complexity proposed by Safford and colleagues to categorize other independent variables associated with complex comorbidities [22]. *Socio-economic variables* consisted of (i) Medicare prescription drug coverage gap; and (ii) insurance status (Private Fee-for-service, Health Maintenance Organization, and other insurance). *Environmental factors* consisted of (i) region (South, Mid-West, and Other regions). The *cultural factors* was defined by race/ethnicity (Whites, African- Americans, Hispanics, and Others). The *behavioral factors* consisted of baseline emergency room visits, and baseline home health visits.

#### Statistical analyses

Unadjusted differences in process and intermediate clinical outcomes among elderly individuals with and without Parkinson’s disease were determined using chi-square tests. Conditional Logistic regression analyses and conditional multinomial logistic regression analyses were conducted for binary dependent variables and dependent variables with more than two levels respectively. As 30 % of the study cohort did not have laboratory values, sample selection models were used to test selection bias among individuals with and without laboratory values for misspecification bias due to non-randomly missing data. This was accomplished using “heckprob” selectivity corrected regression. These models consisted of a selection equation in which the presence or absence of laboratory values were modelled. In the outcome equation, the intermediate clinical outcomes were modelled. For example, for HbA1c control, a logistic regression on the presence or absence of HbA1c values was conducted. In the outcome equation, glycemic control (<8 % and ≥ 8 %) was modelled. The Wald test of independence showed that the chi-square probability value was 0.7968 indicating that there is no influence of unobserved variables on glycemic control outcome in this dataset. Similar findings were observed with the lipid outcomes. Therefore, we report results from analyses among elderly individuals with available HbA1C values and lipid values.

## Propensity score matching

**Fig. 1:**
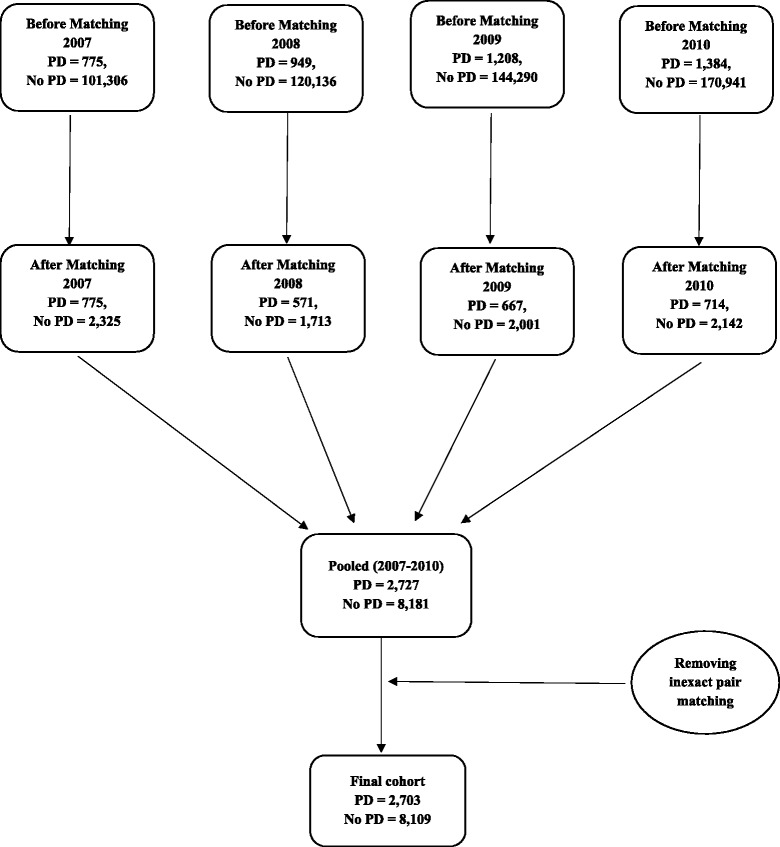
Cohort development

Before propensity score matching, there were 2,727 individuals with PD and diabetes (cases), and 249,763 individuals with diabetes only (controls). After propensity score matching and removing pairs with inexact matches, there were 2,703 individuals with PD and diabetes and 8,109 individuals with diabetes only (1:3 case to control matching). The cohort development is depicted in Fig. 1. Age and Diabetes Complications Severity Index (DCSI) total were continuous variables and the group differences in these two variables were ascertained by t-tests. For gender the group differences were ascertained by using chi-square. The number of individuals with PD before and after matching in each year are different because of the fact that we found that 1,282 individuals without PD in previous years were diagnosed with PD in the following year, and as these individuals were included in the control dataset for a particular year, they were deleted from the control dataset so that we do not have overlap between case and control group during matching. For each year, the individuals with PD in the matched sample were unique cases.

The two groups were matched on age, gender and DCSI scores. The c-statistics of the logistic regression to calculate propensity score for each year were found to be satisfactory (around 0.70). From Tables 1 and 2, it can be noted that, before propensity score matching, the two groups differed significantly from each other in terms of age, DCSI total and gender distribution. Before matching, among individuals with T2DM and PD, the total mean DCSI score was significantly higher compared to those with only diabetes. In terms of age, individuals with PD and diabetes had a significantly higher mean age as compared to those with diabetes only prior to propensity score matching. The PD and diabetes group had significantly higher number of males as compared to the group with diabetes only before matching. The propensity score matched sample was found to be well balanced in terms of the variables that were used to match the two groups. After matching there were no significant differences between the two groups in terms of age, DCSI and gender. From the bottom panel of Tables 1 and 2, it can be noted that when the 4 panels (2007–2008, 2008–2009, 2009–2010, and 2010–2011) were stacked, there were no statistically significant group differences in terms of age, gender and DCSI.

**Table 1:** Distribution of matching variables before and after Propensity Score Matching (PSM) in each year

		Before Matching			After Matching		
		2007					
		PD	No PD		PD	No PD	
		*N* = 775	*N* = 101,306	Sig	*N* = 775	*N* = 2,325	Sig
Age (Mean ± SD)		74.57 (±4.96)	72.56 (±5.02)	***	74.57 (±4.96)	74.56 (±4.95)	
DCSI Total (Mean ± SD)		3.42 (±2.38)	2.22 (±2.11)	***	3.42 (±2.38)	3.42 (±2.38)	
Gender				***			
	Male (N, %)	461 (59.48 %)	47,844 (47.23 %)		461 (59.48 %)	1,385 (59.57 %)	
	Female (N, %)	314 (40.52 %)	53,462 (52.77 %)		314 (40.52 %)	940 (40.43 %)	
		2008					
		PD	No PD		PD	No PD	
		*N* = 949	*N* = 120,136	Sig	*N* = 571	*N* = 1,713	Sig
Age (Mean ± SD)		75.16 (±5.18)	72.80 (±5.19)	***	74.95 (±5.32)	74.94 (±5.32)	
DCSI Total (Mean ± SD)		3.46 (±2.34)	2.31 (±2.15)	***	3.42 (±2.33)	3.42 (±2.34)	
Gender				***			
	Male (N, %)	535 (56.38 %)	56,985 (47.43 %)		307 (53.77 %)	919 (53.65 %)	
	Female (N, %)	414 (43.62 %)	63,151 (52.57 %)		264 (46.23 %)	794 (46.35 %)	
		2009					
		PD	No PD		PD	No PD	
		*N* = 1,208	*N* = 144,290	Sig	*N* = 667	*N* = 2,001	Sig
Age (Mean ± SD)		75.24 (±5.48)	73.00 (±5.37)	***	74.54 (±5.61)	74.59 (±5.60)	
DCSI Total (Mean ± SD)		3.59 (±2.38)	2.44 (±2.19)	***	3.57 (±2.47)	3.54 (±2.41)	
Gender				***			
	Male (N, %)	710 (58.77 %)	68,650 (47.58 %)		382 (57.27 %)	1,153 (57.62 %)	
	Female (N, %)	498 (41.23 %)	75,640 (52.42 %)		285 (42.73 %)	848 (42.38 %)	
		2010					
		PD	No PD		PD	No PD	
		*N* = 1,384	*N* = 170,941	Sig	*N* = 714	*N* = 2,142	Sig
Age (Mean ± SD)		75.43 (±5.63)	73.20 (±5.48)	***	74.77 (±5.88)	74.79 (±5.87)	
DCSI Total (Mean ± SD)		3.66 (±2.43)	2.49 (±2.21)	***	3.65 (±2.44)	3.64 (±2.42)	
Gender				***			
	Male (N, %)	815 (58.89 %)	80,924 (47.34 %)		416 (58.26 %)	1,250 (58.36 %)	
	Female (N, %)	569 (41.11 %)	90,017 (52.66 %)		298 (41.74 %)	892 (41.64 %)	

Note: This table presents the matching variable distribution before and after propensity score matching by individual year

*DCSI* Diabetes Complications Severity Index

***P < 0.001; **0.001 ≤ *P* < .01; *0.01 ≤ *P* < 0.05

**Table 2:** Distribution of matching variables after PSM

Humana Medicare Advantage Part-D Database (2007–2010 stacked)			
	PD	No PD	
	*N* = 3,665	*N* = 10,995	Sig
Age (Mean ± SD)	74.84 (±5.56)	74.86 (±5.56)	
DCSI Total (Mean ± SD)	3.53 (±2.43)	3.52 (±2.41)	
Gender			
Male (N, %)	2,092 (57.08 %)	6,296 (57.26 %)	
Female (N, %)	1,573 (42.92 %)	4,699 (42.74 %)	

Note: This table is based on propensity score matched data (matched on baseline age, gender and Diabetes Complications Severity Index) from Humana Medicare Prescription-Drug Plan of 10,812 elderly Medicare beneficiaries (2,703 cases with Parkinson’s disease and type-2 Diabetes Mellitus and 8,109 controls with only type-2 Diabetes Mellitus) during the period of January 2007 to December 2011

*DCSI* Diabetes Complications Severity Index

## Results

### Description of study sample by PD status

Table 3 exhibits baseline characteristics of T2DM elderly individuals with and without PD. There was significantly higher proportion of African-Americans (14.4 %) among individuals without PD. Overall, both the groups had higher proportions of whites (75.7 % and 74.3 % of individuals with and without respectively). In terms of region and plan types, there were higher proportion of individuals in the South region (around 75 %) and Health Maintenance Organizations in both the groups (around 48 %). There was significantly higher proportion of individuals who did not reach donut hole (56.6 %) among individuals with only diabetes, whereas the group with PD and diabetes had higher proportions of individuals entering (44.9 %) and having entry and exit information regarding donut hole (17.7 %). Individuals with PD and diabetes had significantly greater proportion of polypharmacy users in the higher quintiles (4–5, and 6–31). With respect to the conditions specific to elderly individuals, individuals with PD and diabetes had significantly higher proportion of urinary incontinence (10.4 %), major depressive disorders (26.7 %), cognitive impairment (33.4 %), and falls and falls risk (9.9 %). Individuals with PD had higher proportion of baseline emergency room (55.5 %) and baseline home health visit (60.9 %).

**Table 3:** Baseline characteristics of Elderly Medicare Beneficiaries after matching

		PD	Col %	No PD	Col %	Sig
Race/Ethnicity						***
	White	2,047	75.7	6,025	74.3	
	African American	293	10.8	1,168	14.4	
	Other	169	6.3	446	5.5	
	Unknown	194	7.2	470	5.8	
Region						***
	South	1,806	66.8	5,223	64.4	
	Midwest	604	22.3	1,778	21.9	
	Other Region	293	10.8	1,108	13.7	
Plan Type						***
	HMO	1,315	48.6	3,648	45.0	
	PFFS	918	34.0	2,536	31.3	
	Others	470	17.4	1,925	23.7	
Donut Hole						***
	No DH	1,012	37.4	4,590	56.6	
	Beg DH	1,213	44.9	2,937	36.2	
	Beg/End DH	478	17.7	582	7.2	
Polypharmacy quintile						***
	0 – 0	357	13.2	1,644	20.3	
	1 – 1	454	16.8	1,749	21.6	
	2 – 3	539	19.9	1,818	22.4	
	4 – 5	620	22.9	1,570	19.4	
	6 - 31	733	27.1	1,328	16.4	
Urinary Incontinence						***
	Yes	280	10.4	353	4.4	
	No	2,423	89.6	7,756	95.6	
Major Depressive Disorder						***
	Yes	722	26.7	963	11.9	
	No	1,981	73.3	7,146	88.1	
Cognitive Impairment						***
	Yes	904	33.4	686	8.5	
	No	1,799	66.6	7,423	91.5	
Dominant Conditions^a^						
	Yes	340	12.6	989	12.2	
	No	2363	87.4	7120	87.8	
Falls and falls risk						***
	Yes	268	9.9	354	4.4	
	No	2435	90.1	7755	95.6	
Baseline ER visit						***
	Yes	1500	55.5	3172	39.1	
	No	1203	44.5	4937	60.9	
Baseline HH visit						***
	Yes	1647	60.9	3736	46.1	
	No	1056	39.1	4373	53.9	

*Note*: Based on propensity score matched data (matched on baseline age, gender and Diabetes Complications Severity Index) from Humana Medicare Prescription-Drug Plan of 10,812 elderly Medicare beneficiaries (2,703 cases with Parkinson’s disease and type-2 Diabetes Mellitus and 8,109 controls with only type-2 Diabetes Mellitus) during the period of January 2007 to December 2010

*PD* Parkinson’s disease, *ER* Emergency Room, *HH* Home Health, *HMO* Health Maintenance Organization, *PFFS* Private Fee for Service, *Sig* Significance, *LDL* Low Density Lipoprotein, *HDL* High Density Lipoprotein, *Trigly* Triglyceride, *chol* Cholesterol

Asterisks represent significant group differences in HbA1c testing according to American Diabetes Association (ADA) guidelines using conditional logistic regression adjusting for the matched pair design

***P < 0.001; **0.001 ≤ *P* < .01; *0.01 ≤ *P* < 0.05

^a^Dominant conditions consisted of cancers, end stage renal disease, end stage liver disease, and amputations

## Bivariate and multivariate analyses of process of care and intermediate clinical outcomes

Table 4 summarizes the findings of bivariate and multivariate analyses in terms of process of care measures and intermediate clinical outcomes. Overall, 66.84 % had ADA recommended HbA1c testing. A lower percentage of individuals with PD received HbA1c testing (63.7 %) compared to those without PD (67.9 %, P-value <0.001). An overwhelming majority of individuals with T2DM received lipid testing (84.65 %). A lower percentage of elderly individuals with PD (80.4 %) received lipid testing compared to those without PD (86.1 %, p-value < 0.001). No statistically significant differences were observed in the two groups in terms of nephropathy screening. Among elderly individuals with available HbA1C values, an overwhelming majority had HbA1C value of < 8 % in both individuals with PD (86 %) and without PD (83.8 %). Elderly individuals with PD had statistically significantly better intermediate clinical outcomes compared to those without PD in terms of LDL-C < 100 mg/dl (75.5 % vs. 69.8 %), triglycerides < 150 mg/dl (63.8 % vs. 58.6 %), total cholesterol < 200 mg/dl (87.2 % vs. 83 %) and HDL-C ≥ 50 mg/dl (39.8 % vs. 35.7 %).

**Table 4:** Bivariate and multivariate analyses of process of care and intermediate clinical outcomes

Humana Medicare Advantage Part D database (2007–2011)											
		PD	Col %	No PD	Col %	Sig					
		*N* = 2,703		*N* = 8,109					AOR^a^	95% CI	Sig
HbA1c Testing						***	HbA1c Testing				
	Yes	1,722	63.70	5,505	67.90			Parkinson	0.88	[0.79,0.97]	*
	No	981	36.30	2,604	32.10			No Parkinson			
Lipid Testing						***	Lipid Testing				
	Yes	2,174	80.40	6,978	86.10			Parkinson	0.82	[0.72,0.94]	**
	No	529	19.60	1,131	13.90			No Parkinson			
Nephropathy Screening							Nephropathy Screening				
	Yes	2,027	75.00	6,015	74.20			Parkinson	0.99	[0.88,1.10]	
	No	676	25.00	2,094	25.80			No Parkinson			
Intermediate Clinical Outcomes among those with Available Lab Values											
		PD	Col %	No PD	Col %	Sig			AOR^b^	95% CI	Sig
HbA1c groups		*N* = 1,247		*N* = 3,736			HbA1c <8%				
	> = 8%	174	14.00	604	16.20			Parkinson	1.34	[1.10,1.63]	**
	<8%	1,073	86.00	3,132	83.80			No Parkinson			
LDL-C groups		*N* = 559		*N* = 1938		**	LDL-C <100 mg/dL				
	> = 100 mg/dl	137	24.50	586	30.20			Parkinson	1.29	[1.06,1.59]	*
	<100 mg/dl	422	75.50	1,352	69.80			No Parkinson			
Triglyceride groups		*N* = 558		*N* = 1977		*	Triglyceride <150 mg/dL				
	> = 150 mg/dl	202	36.20	819	41.40			Parkinson	1.31	[1.06,1.62]	*
	<150 mg/dl	356	63.80	1,158	58.60			No Parkinson			
Total Cholesterol groups		*N* = 561		*N* = 1982		*	Total Cholesterol < 200 mg/dL				
	> = 200 mg/dl	72	12.80	336	17.00			Parkinson	1.46	[1.08,1.97]	*
	<200 mg/dl	489	87.20	1,646	83.00			No Parkinson			
HDL-C groups		*N* = 1157		*N* = 3676		*	HDL-C ≥ 50 mg/dL				
	<50 mg/dl	696	60.20	2,364	64.30			Parkinson	1.20	[1.04,1.39]	*
	> = 50 mg/dl	461	39.80	1,312	35.70			No Parkinson			

*Note*: Based on propensity score matched data (matched on baseline age, gender and Diabetes Complications Severity Index) from Humana Medicare Prescription-Drug Plan of 10,812 elderly Medicare beneficiaries (2,703 cases with Parkinson’s disease and type-2 Diabetes Mellitus and 8,109 controls with only type-2 Diabetes Mellitus) during the period of January 2007 to December 2011 (except the bottom panel)

*PD* Parkinson’s disease, *Sig* Significance, *LDL* Low Density Lipoprotein, *HDL* High Density Lipoprotein, *Trigly* Triglyceride, *yr* Year, *chol* Cholesterol, *AOR* Adjusted Odds Ratio, *CI* Confidence Interval

***P < 0.001; **0.001 ≤ *P* < .01; *0.01 ≤ *P* < 0.05

^a^AOR obtained from conditional logistic regression

^b^AOR obtained from multinomial conditional logistic regression. Reference groups: ≥8% HbA1c; LDL-C ≥ 100 mg/dL; Triglyceride ≥ 150 mg/dL; LDL-C ≥ 50 mg/dL; Total Cholesterol ≥ 200mg/dL

The upper panel of the right hand portion of Table 4 shows the results of conditional logistic regression analyses conducted with HbA1c testing as the dependent variable adjusting for the matched pair design. After controlling for Parkinson’s disease, race/ethnicity, region, plan-type, donut hole, polypharmacy, urinary incontinence, depression, falls and fall risk, cognitive impairment due to physical conditions, cognitive impairment due to mental conditions, dominant conditions, baseline emergency room visits, baseline home health visits and adjusting for the matched pair design, it was observed that individuals with PD were 12 % (AOR: 0.88, 95 % CI: 0.79-0.97) and 18 % (AOR: 0.82, 95 % CI: 0.72-0.94) less likely to meet the annual ADA recommended HbA1c and lipid testing respectively compared to individuals without PD. However, there were no statistically significant difference between individuals with and without PD in terms of nephropathy testing (AOR: 0.99, 95 % CI: 0.88-1.10).

The lower panel of the right hand portion of Table 4 shows the results from conditional multinomial logistic regression with glycemic control as the dependent variable. After adjusting for Parkinson’s disease, race/ethnicity, region, plan-type, donut hole, polypharmacy, urinary incontinence, depression, falls and fall risk, cognitive impairment due to physical conditions, cognitive impairment due to mental conditions, dominant conditions, baseline emergency room visits, baseline home health visits and matched pair design, it was observed that individuals with PD were 34 % (AOR: 1.34, 95 % CI: 1.10-1.63) more likely to have better glycemic control (HbA1c < 8 %) compared to those without PD. Individuals with PD were higher likely to have better outcomes in terms of LDL-C (<100 mg/dl) (AOR: 1.29, 95 % CI: 1.06-1.59), triglyceride (<150 mg/dl) (AOR: 1.31, 95 % CI: 1.06-1.62), total cholesterol (<200 mg/dl) (AOR: 1.46, 95 % CI: 1.08-1.97) and HDL-C (≥50 mg/dl) (AOR: 1.20, 95 % CI: 1.04-1.39).

## Discussion

This study examined the association between PD and process and intermediate clinical outcomes of diabetes care among elderly individuals with T2DM. Results from this study indicated that among individuals with T2DM, those with PD did not receive the ADA recommended HbA1c and lipid testing compared to those without PD. These findings suggest that, PD is a barrier to achieving clinically recommended process of care measures. We can speculate the reasons as to why those with PD did not achieve the ADA recommended HbA1c and lipid testing. Some of the reasons which may lead to not achieving the ADA recommended HbA1c and lipid testing can be that elderly Medicare beneficiaries may not be aware of the benefits of meeting these goals, or there can be a gap in patient-provider communication, or the elderly Medicare beneficiaries may not be visiting their physicians on a regular basis [23]. Some of the ways in which these barriers can be overcome include educating patients and helping them to attend group consultations by healthcare providers [24]. Future studies should explore the possible reasons why elderly individuals with co-occurring T2DM and PD are not being able to achieve the ADA recommended process of care goals.

A noteworthy finding from our study is the relationship between PD and intermediate clinical outcomes of diabetes care. For glycemic and lipid outcomes, elderly individuals with PD were more likely to achieve control compared to those without PD. A plausible explanation for better outcomes among those with PD could be due to pathophysiological conditions of the two diseases. For example, it has been suggested that insulin resistance and insulin deficiency, which are the cardinal characteristics of T2DM, can lead to neurodegeneration [9–11]. It is possible that given the risk of neurodegeneration due to T2DM, the providers may be aggressively treating individuals with T2DM and PD for better glycemic and lipid outcomes in order to prevent further neurodegeneration. The findings from this study are consistent with a study conducted among elderly veterans, which showed that veterans with PD were more likely to receive overall good outcomes for all three intermediate clinical outcomes - glycemic, blood pressure and low density lipoprotein-cholesterol combined (adjusted OR, 2.17; 95 % CI, 1.96-2.39) [6]. One of the similarities between VA and the Medicare Advantage (MA) plans is that they follow the Integrated Delivery System (IDS) model or the “coordinated care plan” approach. In the IDS model, the primary physician serves as the gate-keeper and maintains proper referral systems. The coordination of care among different types of specialists (Endocrinologists, Neurologists etc.) is ensured in the IDS models which in turn could lead to better management of individuals with PD. One can speculate that such coordination may involve comprehensive geriatric assessment (CGA) tools, which in turn can lead to better intermediate clinical outcomes among elderly beneficiaries with T2DM and PD.

However, the findings from this study is inconsistent with the findings from the study using the INTERMED classification system for case complexity which found that among individuals with T2DM, greater complexity was associated with higher HbA1c values [7]. The INTERMED classification system utilized different factors such as biological, psychosocial and health care related aspects of T2DM to classify patient complexity and had only 61 patients in the study. The results from this study [7] presented were in the preliminary forms and it required further validation. Hence, it is possible that due to different classification system used to determine patient complexity, greater risk of poor diabetes control among individuals with higher complexity was observed.

## Strengths and limitations

Strengths of this study include the use of large sample size, nationwide sample of commercially insured elderly individuals, exhaustive list of variables, availability of laboratory values and use of a robust study design.

As with other studies, this study also has limitations. Findings from this study cannot be generalizable to other populations or settings (e.g., fee-for-service Medicare beneficiaries). Laboratory values are available for only one-third of the population. Unmeasured confounders such lifestyle risk factors (e.g., body mass index and smoking status), physician specialty, duration and severity of PD could influence the study outcomes.

## Conclusion

To the best of author’s knowledge, this is the first study to examine the influence of the presence of a chronic illness with complexity such as PD on the process and outcomes of diabetes care. Individuals with PD and diabetes were less likely to achieve ADA recommended annual HbA1c and lipid testing goals compared to those with diabetes but without PD. Future research needs to explore the reasons for lower rates of HbA1C and lipid testing among elderly individuals with T2DM and PD. However, among individuals with PD, the intermediate glycemic and lipid outcomes were better compared to those without PD. These findings suggest that the integrated delivery system coupled with the CGA approach of MA plans may be beneficial to elderly with PD.

## Appendix

### HbA1c testing

Individuals with HbA1c testing at least two times a year (with a gap of at least one month) will be considered as meeting standard of care for diabetes management. HbA1c testing will be identified by the following Current Procedural Terminology (CPT) codes.

- CPT codes: 83036, 83037 (Source : HEDIS, 2012)
- CPT Category II : 3044F, 3045F, 3046F, 3047F (Source : HEDIS, 2012)

### Lipid testing

Individuals who were tested at least once a year will be considered as meeting standard of care for diabetes management. Lipid testing will be identified by the following Current Procedural Terminology (CPT) codes.

- CPT codes: 80061, 83700, 83701, 83704, 83721, 83715, 83716, 83718, 82465, and 84478 (Source: HEDIS, 2012)
- CPT Category II: 3048F, 3049F, 3050F

### Nephropathy screening

Individuals who were screened at least once a year for urine albumin and serum creatinine will be considered as meeting standard of care for diabetes management. Nephropathy screening will be identified using the following Current Procedural Terminology (CPT) codes.

- CPT codes: 81000, 81001, 81002, 81003, 36800, 36810, 36815, 50300, 50340, 50360, 50365, 50370, 50380, 90920, 90921, 90924, 90925, 90935, 90937, 90945, 90947, 90989, 90993, 90997, and 90999 82579 for serum creatinine lab; 82042, 82043, 82044, 84156 (Source: HEDIS, 2012)
- CPT Category II: 3060F, 3061F (Source: HEDIS, 2012)
